# Adenosine deaminase mediates endothelial inflammation via an ADA1-CD26 interaction in post-COVID 

**DOI:** 10.3389/fphar.2025.1578973

**Published:** 2025-05-21

**Authors:** Ada Kawecka, Klaudia Stawarska, Marzena Romanowska-Kocejko, Marta Żarczyńska-Buchowiecka, Agata Jędrzejewska, Alicja Braczko, Milena Deptuła, Małgorzata Zawrzykraj, Oliwia Król, Marika Frańczak, Gabriela Harasim, Michał Pikuła, Marcin Hellmann, Barbara Kutryb-Zając

**Affiliations:** ^1^ Department of Biochemistry, Medical University of Gdansk, Gdańsk, Poland; ^2^ Department of Cardiac Diagnostics, Medical University of Gdansk, Gdańsk, Poland; ^3^ Laboratory of Tissue Engineering and Regenerative Medicine, Division of Embryology, Medical University of Gdansk, Gdańsk, Poland; ^4^ Division of Clinical Anatomy, Department of Anatomy, Medical University of Gdansk, Gdańsk, Poland

**Keywords:** adenosine deaminase, CD26, post-COVID-19 syndrome, endothelium, immune cells

## Abstract

**Introduction:**

Adenosine deaminase (ADA) isoenzymes play a role in microvascular dysfunction following SARS-CoV-2 infection. This study analyzes the mechanisms behind ADA1-dependent endothelial inflammation in post-COVID-19 syndrome. We investigated whether immune cells from post-COVID patients could contribute to the increased total ADA activity. Additionally, we examined ADA’s enzymatic and extra-enzymatic activities in human primary lung microvascular endothelial cells (HULECs) stimulated with post-COVID patients’ serum.

**Methods and results:**

Treatment of HULECs with sera from post-COVID patients resulted in elevated levels of the ADA1 isoenzyme and the ADA1-anchoring protein, CD26. This increase correlated with enhanced adhesion of THP-1 monocytes/macrophages to HULECs. Inhibiting the ADA1-CD26 interaction with glycoprotein-120 prevented the rise in cell-surface ADA levels in HULECs and reduced the adhesion of THP-1 cells to the endothelium. A similar effect was observed when HULECs were pre-incubated with the SARS-CoV-2 spike protein, which co-localized with CD26 in activated HULECs.

**Conclusions:**

We propose that ADA1 promotes vascular inflammation in post-COVID-19 syndrome through both canonical and non-canonical mechanisms. On one hand, its increased enzymatic activity can suppress adenosine-dependent pathways. On the other hand, ADA1 may function as an adhesion molecule facilitating interactions between immune cells and the endothelium via ADA1-CD26 complexes.

## Graphical Abstract

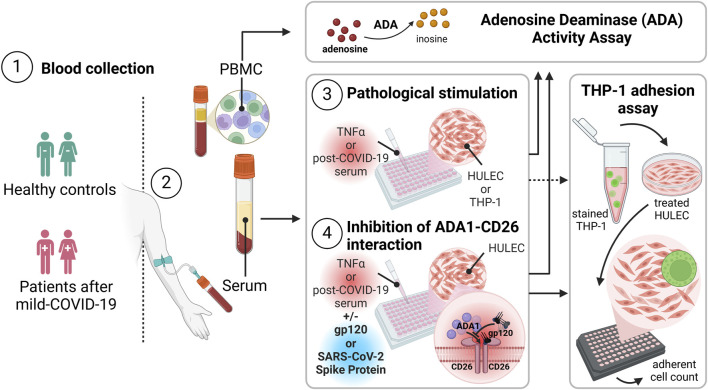

## 1 Introduction

Accumulating data suggest a pivotal involvement of purinergic signaling pathways in the microvascular dysfunction caused by coronaviral diseases. These pathways modulate the immune response, endothelial function, and platelet reactivity, critical for vascular homeostasis, inflammation, and thrombosis processes (Franciosi et al., 2021). The disruption of cellular metabolism by coronaviral invasion leads to the release of endogenous danger molecules, such as nucleotides from infected cells (Ribeiro et al., 2021). These molecules, referred to as danger-associated molecular patterns (DAMPs) or pathogen-associated molecular patterns (PAMPs), accumulate at sites of tissue injury and inflammation, exacerbating the pathological processes (Naqvi et al., 2022). For instance, it has been shown that extracellular adenosine triphosphate (ATP), acting via P2X and P2Y purinoreceptors, triggers inflammatory activation of endothelial cells and follows microvascular dysfunction (Cardoso et al., 2017).

In addition to receptor stimulation, ATP undergoes extracellular hydrolysis by the cell surface ecto-nucleoside triphosphate diphosphohydrolase 1 (CD39/ENTPD1), resulting in adenosine diphosphate (ADP) formation, which is further converted to adenosine monophosphate (AMP). AMP is then hydrolyzed to adenosine by ecto-5′-nucleotidase (CD73/NT5E) (Kochan et al., 1994; Smolenski et al., 1992). Both ecto-nucleotides are critical for proper endothelial cell function (Mierzejewska et al., 2019; Chisci et al., 2017). This depends on adenosine, which activates intracellular signaling cascades upon binding to a family of G protein-coupled receptors: A1, A2a, A2b, and A3, abundantly expressed in various tissues and cell types (Sheth et al., 2014). It has been well described that adenosine counteracts endothelial inflammation and improves endothelial barrier function via receptor mechanisms. Adenosine bioavailability is regulated by its production, cellular uptake by nucleoside transporters, and catabolism. Intracellularly, adenosine is maintained at low concentrations by adenosine kinase (AK) and adenosine deaminase (iADA). Meanwhile, extracellular adenosine undergoes vigorous deamination by cell surface (ecto-ADA) or soluble (sADA) adenosine deaminase (Kutryb-Zajac et al., 2020a). There are two ADA isoenzymes, ADA1 and ADA2. Our previous studies demonstrated that ADA1 is ubiquitously expressed in the vascular cells, including the endothelium, while ADA2 originates only from monocytes/macrophages (Kutryb-Zajac et al., 2019). Additionally, we revealed an increased activity of endothelial cell-surface ADA1 in cardiovascular diseases such as atherosclerosis (Kutryb-Zajac et al., 2014) and aortic valve stenosis (Kutryb-Zajac et al., 2020b). Besides a well-established catalytic role, adenosine metabolism ecto-enzymes may exhibit non-canonical, extra-enzymatic functions (Airas et al., 1995; Cortés et al., 2015). Recent evidence demonstrates that extracellular ADA1, through binding to dipeptidyl peptidase-4 (CD26) and adenosine receptors (AR), forms complexes serving as adhesion molecules via CD26-ADA1-AR. This becomes particularly noteworthy in cell-to-cell interactions between endothelium and blood cells, playing a role in developing vascular pathologies (Carman and Martinelli, 2015; Coenen et al., 2017; Wautier and Wautier, 2020). Our previous study elucidated a potential interplay between ADA isoenzymes and COVID-19 pathophysiology (Jedrzejewska et al., 2023). We established a correlation between the increased serum ADA2 activity and endothelial and microvascular dysfunction in post-COVID patients. In the present study, we aimed to analyze the mechanisms of endothelial inflammation mediated by adenosine deaminase.

## 2 Materials and methods

### 2.1 Human participants

All participants provided written informed consent following the principles outlined in the Declaration of Helsinki. The research protocol received approval from the Independent Bioethics Committee for Scientific Research at the Medical University of Gdansk, Poland, under the license numbers NKBBN/55/2021 and KB/547/2024. Blood specimens were acquired from individuals who had experienced persistent cardiovascular-related symptoms, including fatigue, palpitations, dyspnea, chest pain, or tachycardia, 12 weeks after a positive PCR test for SARS-CoV-2 (post-COVID). Healthy controls had no previous diagnosis of SARS-CoV-2 infection (Controls) as detailed in Table 1. The sample collection took place in February 2021. None of the participants had received any vaccine doses. Peripheral blood was centrifuged at 1,200 x g for 15 min at room temperature to obtain the serum, frozen at −80°C for subsequent analyses. Peripheral blood mononuclear cells (PBMC) were isolated from blood collected with EDTA using the Histopaque protocol. Briefly, the collected blood was diluted with an equal volume (1:1) of 0.9% saline solution and placed over 3 mL of Histopaque-1077 on top of Histopaque-1119 in a Falcon tube. The tube was centrifuged at room temperature at 500 x g for 30 min. Then, the cloud of mononuclear cells (mainly lymphocytes and monocytes) made between the plasma and Histopaque-1077 layer was aspirated and transferred to another tube, which was washed thrice with 0.9% saline and maintained until use at −80°C.

**TABLE 1:** Patient characteristics. Healthy controls (Control, n = 5) and post-COVID patients (Post-COVID, n = 5) characteristics. Results are shown as mean ± SEM. *p < 0.05, **p < 0.01. F–female, M–male, Na–not applicable, hsCRP–high sensitive C-reactive protein, TNFα–tumor necrosis factor alpha, IL-10 – interleukin 10, sICAM-1 – soluble intercellular adhesion molecule 1, NLR–neutrophil-to-lymphocyte ratio, LCR–lymphocyte-to-C-reactive protein ratio, LMR–lymphocyte-to-monocyte ratio, PLR–platelet-to-lymphocyte ratio.

	Control	Post-COVID
Age [years]	43 ± 4	44 ± 5
Gender (F/M)	3/2	3/2
SARS-CoV-2 PCR (+)	Na	Dec 2020
Sample collection	February 2021	February 2021
post-COVID symptoms		
fatigue	0/5	5/5
headaches	0/5	2/5
dyspnea	0/5	1/5
chest pain	0/5	1/5
hsCRP [mg/l]	1.72 ± 0.20	1.88 ± 0.07
TNFα [pg/ml]	5.23 ± 0.89	10.1 ± 0.57**
IL-10 [pg/ml]	3.45 ± 2.00	2.09 ± 0.41
sICAM-1 [ng/ml]	283 ± 48.2	537 ± 154
NLR	1.52 ± 0.15	1.88 ± 0.11
LCR	1.21 ± 0.11	0.89 ± 0.09
LMR	4.81 ± 0.21	3.80 ± 0.19**
PLR	93.5 ± 5.19	114 ± 5.65*

### 2.2 Measurement of serum hs-CRP, sICAM-1, and cytokines

The serum high-sensitive C-reactive protein (hs-CRP) concentration was measured using an Automated Photometer (ERBA XL-180, Mannheim, Germany) and specific ERBA kits according to the manufacturer’s instructions. Serum soluble ICAM-1 (sICAM-1) concentration was measured using an ELISA kit according to the manufacturer’s protocol (Merck, Darmstadt, Germany). TNFα and IL-10 concentrations were measured in serum samples by the Luminex Multiplex platform using dedicated kits (Merck, Darmstadt, Germany) according to the manufacturer’s instructions.

### 2.3 Cell culture and treatment

Human microvascular lung endothelial cells (HULEC-5a, HULECs) were obtained from ATCC (Manassas, VA, United States) and cultured in MCDB131 medium supplemented with 10% fetal bovine serum (FBS), 10 ng/mL Epidermal Growth Factor (EGF), 1 μg/mL hydrocortisone, 10 mM L-glutamine, and 1% penicillin/streptomycin. Human monocytes/macrophages (THP-1) were obtained from ECACC (Salisbury, UK) and cultured in RPMI 1640 medium supplemented with 2 mM L-glutamine, 10% fetal bovine serum, and 1% penicillin/streptomycin. All cultured cells were maintained at 37°C, 5% CO_2_. For the treatments, HULEC cells were seeded at 24-well plates (5 × 10^4^ cells/well) or 96-well plates (0.8 × 10^4^ cells/well), and after reaching 80% confluence cells were washed 3 times with PBS to discard any remaining traces of FBS form the initial culture media, and then incubated for 48h with MCDB131 medium free of FBS supplemented with 20% serum obtained from post-COVID patients (N = 5) or healthy controls (N = 5). In the additional experiments, HULECs were treated for 48h in full medium with 10 ng/mL recombinant TNFα (cat. no. SRP2102, Merck, Germany). Cells were stimulated in the presence or absence of 1.2 μg/mL and 6 μg/mL recombinant glycoprotein-120 (gp-120, cat. no. SAE0071, Merck, Germany) or 5 μg/mL and 10 μg/mL recombinant SARS-CoV-2 spike protein S1 subunit (cat. no. SAB5700591, Merck, Germany) added 30 min before serum or TNFα. After that, HULECs were washed 3 times with PBS and used for further experiments. THP-1 cells were treated at 6-well plates at a density of 1 × 10^6^ cells/well with 10 ng/mL TNFα for 48h in full cell culture medium or with FBS-free medium supplemented with 20% serum obtained from post-COVID patients (N = 5) or healthy controls (N = 5).

### 2.4 Measurement of adenosine deaminase activity on the cell surface and in cell homogenates

HULEC monolayer was treated as described above at 24‐well plates and rinsed with PBS. Then, 1 mL of Hanks Balanced Salt Solution (HBSS) was added to each well. To measure cell-surface total adenosine deaminating activity, 50 μM adenosine (final concentration) was added, and samples were collected after 0, 5, 15, and 30 min of incubation at 37°C and analyzed with UHPLC as described previously (Zukowska et al., 2017). THP-1 cells after the treatment and PBMC were washed with PBS, 500 μL of cold deionized H_2_O was added to each well, and the plates were immediately frozen at −80°C. After thawing, the cell suspension was sonicated for 30 s on ice (30% amplitude, 0.4-s pulse cycle), and total adenosine deaminase activity was measured in the cell homogenate as described previously (Kutryb‐Zajac et al., 2018). Briefly, the reaction was started by the addition of diluted cell homogenate with 50 mmol/L Tris/HCl, pH 7.0 (1:2 v/v) to the incubation buffer (1:1 v/v) containing 50 mmol/L Tris/HCl (pH 7.0), 1 mmol/L adenosine. After the incubation for 15 min at 37°C in constant shaking, the enzymatic reaction was terminated by adding 1.3 mol/L HClO_4_ (1:1 v/v). Then, samples were incubated on ice for 10 min and centrifuged (20,800 g, 10 min, 4°C). Supernatants were brought to pH 5.5–6.5 with 3 mol/L K_3_PO_4_, and UHPLC was used to analyze the concentration of adenosine and inosine in supernatants after the following centrifugation (20,800 g, 10 min, 4°C). Protein concentration was measured in all cells after dissolving the cell residue in 0.5 M NaOH by the Bradford method according to the manufacturer’s protocol. The results were expressed as the inosine increase over time [nmol/min/mg prot].

### 2.5 Fluorescence analysis

HULEC cells treated at 96‐well optical plates (Corning, NY, United States) were rinsed with PBS, and then immunofluorescent staining of ADA1 and CD26 protein was performed. Firstly, the cells were fixed with 4% formalin in PBS for 30 min, and without the permeabilization step, cells were washed twice with PBS. After that, the cells were incubated with a blocking solution of 10% goat serum and 1% BSA in PBS. Then, cells were incubated with primary mouse anti-ADA1 (cat. no. MA5-24586, Thermo Fisher Scientific, Waltham, MO, United States), rat anti-CD26 (cat. no. GTX54493, GeneTex, Hsinchu City, Taiwan), or rabbit anti-SARS-CoV-2 Spike S1 (cat. no. SAB3501119, cat. no. Merck, Germany) antibodies in PBS for 1h. After washing with PBS, Alexa Fluor 488 goat-anti-mouse, Alexa Fluor 594 goat-anti-rabbit, or Alexa Fluor 594 goat-anti-rat secondary antibodies (Jackson Immuno, Cambridgeshire, UK) were added for 30 min. After washing with PBS, cell nuclei were counterstained with 4′,6-diamidino-2-phenylindole (DAPI). Images were taken and analyzed using Axio Observer seven fluorescence microscope and ZEN software.

### 2.6 Immune cell adhesion assay

To study THP-1 cells’ adhesion to endothelial cells, HULEC cells were seeded at 24-well cell imaging plates (Merck Millipore) and treated as described above. After washing with PBS, carboxyfluorescin succinimidyl ester (CFSE)-labeled THP-1 cells (4.5 × 10^4^) were added at 300 μL of FBS-free medium. Cells were co-incubated for 30 min at 37°C, 5% CO_2_, non-adherent THP-1 cells were removed, and plates were rinsed twice with 1 mL FBS-free medium. Then, THP-1 cell adhesion to endothelial monolayers was measured by counting cells with residual green fluorescence staining using Axio Observer seven fluorescence microscope and ZEN software (Carl Zeiss Inc.).

### 2.7 Statistical analysis

Statistical analysis was performed using InStat software (GraphPad, San Diego, CA). Normality was assessed using the Kolmogorov-Smirnov, Shapiro-Wilk, or D'Agostino and Pearson Omnibus tests. Comparison of mean values between groups was evaluated by one-way ANOVA followed by Holm-Sidak or Dunn’s *post hoc* test and by unpaired Student’s t-test or Mann-Whitney test. The exact value of n was provided for each type of experiment. Statistical significance was assumed at p < 0.05. Error bars indicated the standard error of the mean (SEM).

## 3 Results

### 3.1 Total ADA is overactivated in the immune cells of post-COVID patients

**FIGURE 1:**
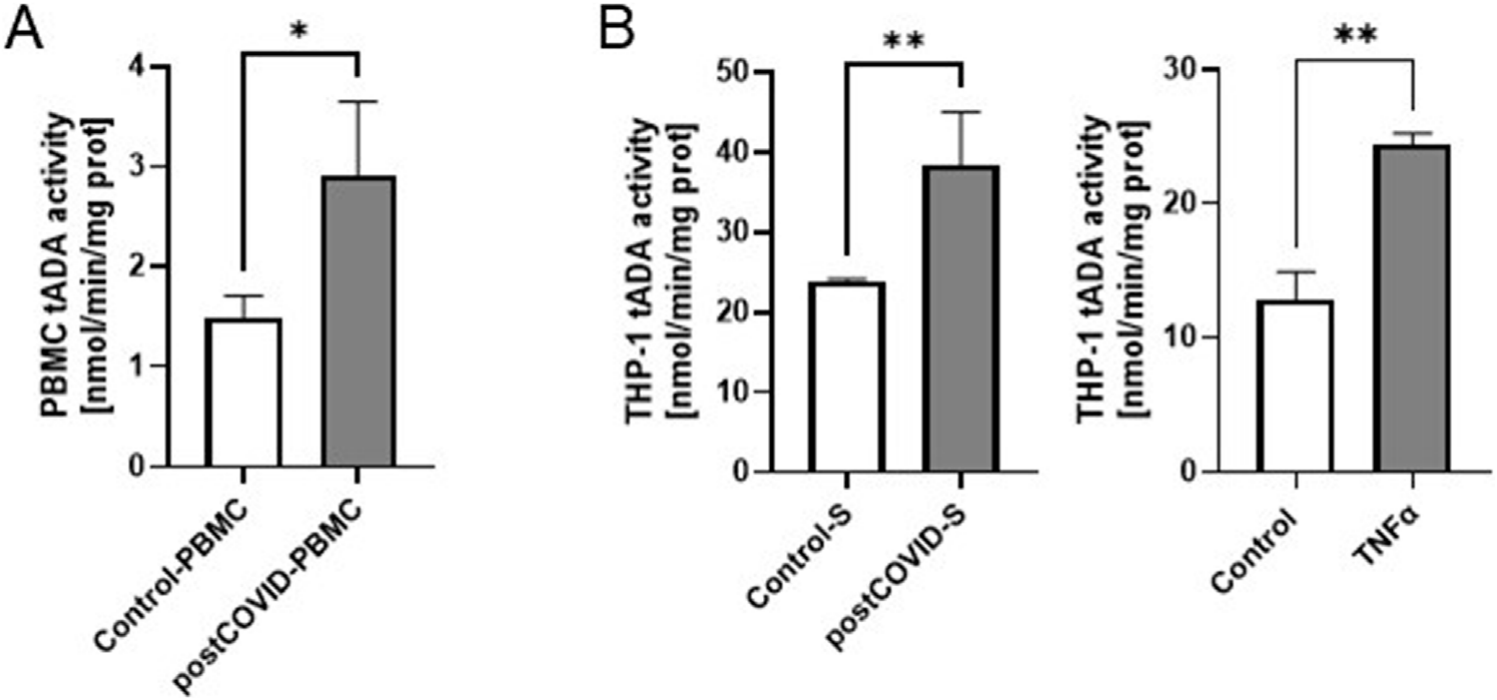
Total adenosine deaminase (tADA) activity in peripheral blood mononuclear cells is higher in post-COVID patients than in healthy controls, and it is enhanced in monocytes/macrophages after incubation with post-COVID serum. **(A)** tADA activity in the homogenates of peripheral blood mononuclear cells (PBMC) isolated from post-COVID patients (n = 5) and healthy controls (n = 5). **(B)** tADA activity in the homogenates of monocytes/macrophages (THP-1) treated for 48 h with. control (Control-S, N = 5 independent experiments with five different patients’ sera, n = 3 biological repetitions for each patient) and post-COVID patients’ (postCOVID-S, N = 3 independent experiments with three different patients’ sera, n = 3 biological repetitions for each patient) sera or vehicle (control, n = 4) and 10 ng/mL TNFα (n = 4). Results are shown as mean ± SEM. *p < 0.05, **p < 0.01.

Post-COVID patients demonstrated higher serum concentration of TNFα and a tendency (p = 0.15) to higher levels of sICAM-1 (Table 1). In addition, we revealed higher lymphocyte-to-monocyte (LMR) and platelet-to-lymphocyte (PLR) ratios. In contrast, neutrophil-to-lymphocyte (NLR) and lymphocyte-to-C-reactive protein (LCR) ratios were slightly affected in post-COVID individuals. In peripheral blood mononuclear cells isolated from post-COVID patients, we have shown higher activity of total ADA than in control PBMC (Figure 1A). It should be noted that rates of adenosine deamination measured in immune cell extracts covered both intracellular and cell-surface ADA activities. Still, our previous study revealed a minor role in immune cell-derived ecto-ADA activity (Kutryb-Zajac et al., 2021). Subsequently, we measured ADA activity in extracts of THP-1 cells (monocytes/macrophages) and revealed that post-COVID serum induced a rise in total ADA (Figure 1B). Similarly, we observed an increase in total ADA activity after the treatment with TNFα (Figure 1B).

### 3.2 Adhesion of immune cells to the activated endothelium is mediated by ADA1-CD26 interaction

**FIGURE 2:**
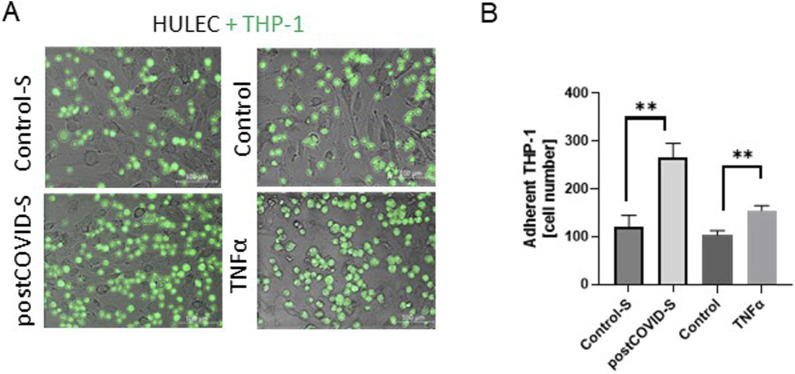
Adhesion of monocytes/macrophages to the lung microvascular endothelial cells is increased in the presence of post-COVID patient sera. **(A)** Representative images and **(B)** quantitative analysis of the adhesion of monocytes/macrophages (THP-1) to primary lung microvascular endothelium (HULEC) pre-treated for 48h with control (Control-S, N = 5 independent experiments with five different patients’ sera, n = 3 biological repetitions for each patient) and post-COVID patient serum (postCOVID-S, N = 5 independent experiments with five different patients’ sera, n = 3 biological repetitions for each patient) or vehicle (control, n = 6) and 10 ng/mL TNFα (n = 6). Results are shown as mean ± SEM, **p < 0.01; Each image includes a scale bar (bottom right) indicating 100 µm.

Then, we analyzed the effect of lung microvascular endothelial cell pre-stimulation with post-COVID sera on THP-1 adhesion. Incubation of HULEC monolayer with patients’ sera for 48h led to a higher degree of THP-1 cell adhesion than the incubation with healthy control sera (Figure 2). A similar trend was noticed when comparing the adhesion of immune cells to TNFα-stimulated HULECs (Figure 2).

**FIGURE 3:**
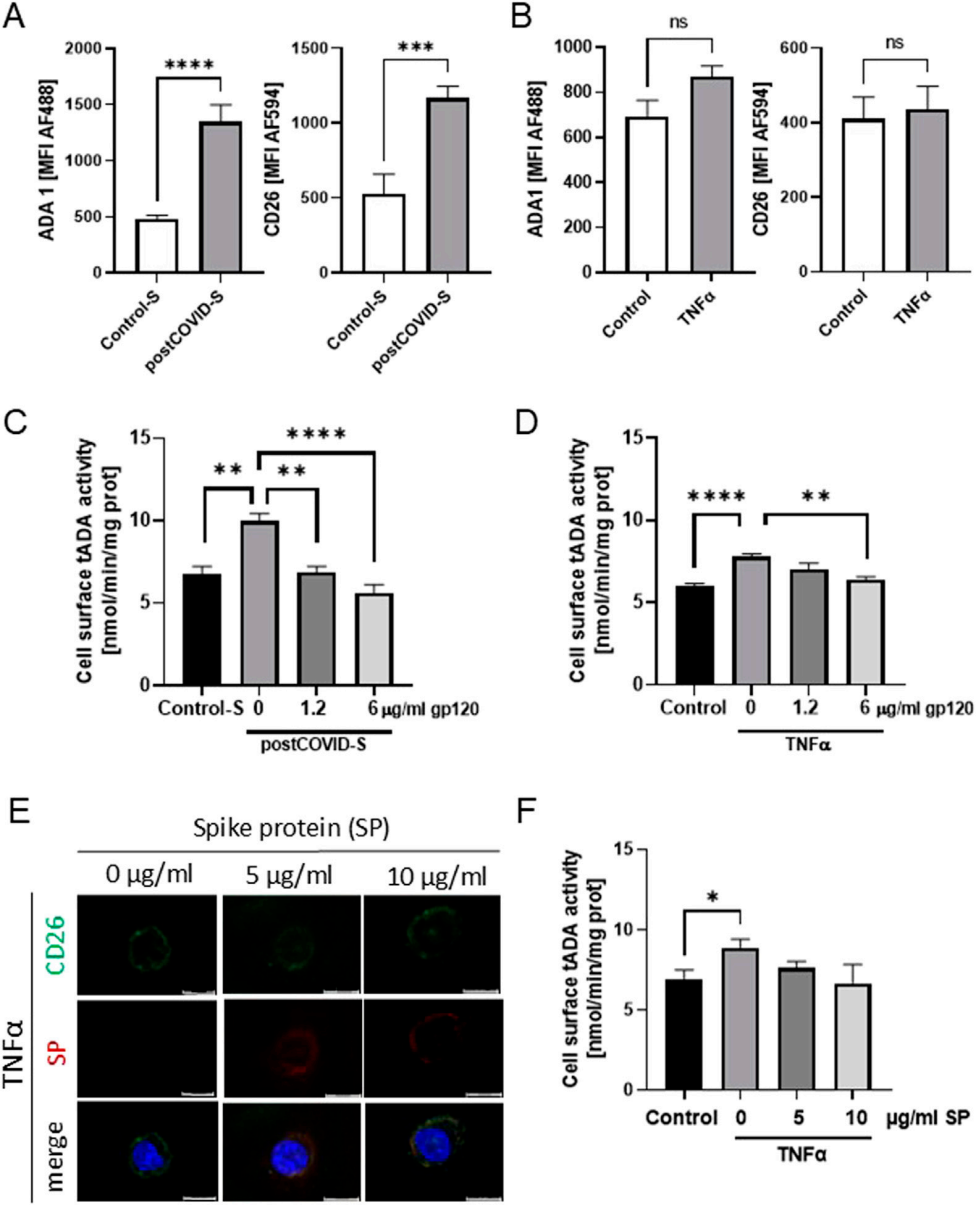
ADA1-CD26 interaction inhibitor, glycoprotein 120 (gp120), prevented the increase in tADA activity on the surface of activated lung microvascular endothelial cells. Mean fluorescence intensity (MFI) for green (AF488) and red (AF594) fluorescence that corresponded to ADA1 (AF488) and CD26 (AF594) in primary lung microvascular endothelial cells (HULEC) treated for 48h with **(A)** control (Control-S, N = 5 independent experiments with five different patients’ sera, n = 3 biological repetitions for each patient) and post-COVID patients’ sera (postCOVID, N = 5 independent experiments with five different patients’ sera, n = 3 biological repetitions for each patient), or with **(B)** vehicle (Control, n = 5) and 10 ng/mL TNFα (n = 5). **(C)** Cell-surface total ADA (tADA) activities in HULEC pre-treated for 30 min with 0, 1.2, or 6 μg/mL gp120 and then treated for 47.5h with **(C)** control (Control-S, N = 5) and post-COVID patients’ (postCOVID-S, N = 5) sera, or with **(D)** vehicle (Control, n = 5) and 10 ng/mL TNFα (n = 5). **(E)** Representative images for immunofluorescence staining of CD26 (AF488, green), SARS-CoV-2 spike protein 1 (SP, AF647, red), cell nuclei (DAPI, blue) in HULEC pre-treated for 30 min with 0, five or 10 μg/mL SP then treated for 47.5h with 10 ng/mL TNFα. **(F)** Cell-surface tADA activity in control pre-treated HULEC for 30 min with 0, 5, or 10 μg/mL SP and then treated for 47.5h with 10 ng/mL TNFα (n = 5 per group). Results are shown as mean ± SEM. *p < 0.05, **p < 0.01, ***p < 0.001, ****p < 0.0001. Each image includes a scale bar (bottom right) indicating 20 µm.

Next, we revealed a higher abundance of ADA1 protein, corresponding to a significant endothelial ADA activity, in HULECs after stimulation with post-COVID sera (Figure 3A). The level of ADA1-anchoring protein, CD26, was also higher after HULEC stimulation with patients’ sera. Similar trends to higher levels of these proteins were observed in HULECs after TNFα treatment (Figure 3B). We also revealed increased cell-surface tADA activity on HULECs after treatment with both post-COVID sera and TNFα (Figures 3C, D). These observations align with our prior findings, elucidating the pro-inflammatory consequences of SARS-CoV-2 infection on the endothelium (Jedrzejewska et al., 2023).

**FIGURE 4:**
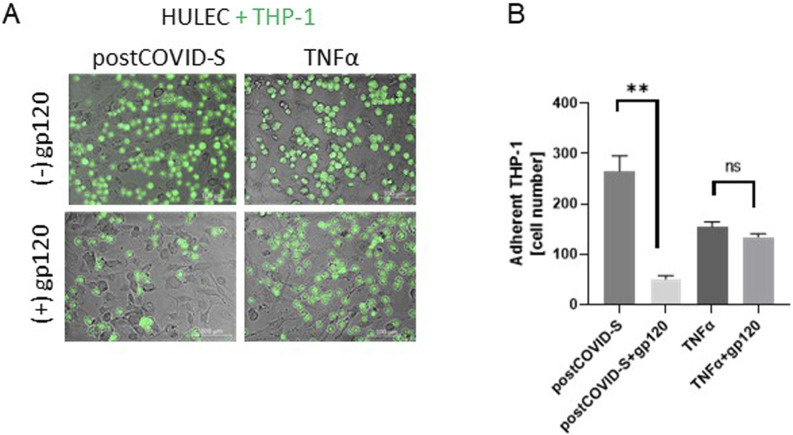
Glycoprotein 120 (gp120), counteracted the increased adhesion of monocytes/macrophages to activated lung microvascular endothelial cells. **(A)** Representative images and **(B)** quantitative analysis of the adhesion of monocytes/macrophages (THP-1) to primary lung microvascular endothelial cells (HULEC) pre-treated for 30 min with 1.2 μg/mL gp120 and then treated for 47.5h with control (Control-S) and post-COVID patients’ (postCOVID-S) sera, or vehicle (Control) and 10 ng/mL TNFα. N = 5 independent experiments with five different patients’ sera, n = three to four biological repetitions for each patient (Control-S, post-COVID-S), n = 6–10 (control, TNFα). Results are shown as mean ± SEM. *p < 0.05, **p < 0.01. Each image includes a scale bar (bottom right) indicating 100 µm.

To study the role of ADA1-CD26 interaction in the pro-inflammatory potential of endothelial cells induced by post-COVID sera exposition, we used recombinant glycoprotein 120 (gp-120), which is known to inhibit the formation of ADA1-CD26 complexes (Valenzuela et al., 1997). We assessed cell-surface total ADA activity and analyzed the adhesion potential of THP-1 cells to HULECs (Figures 3, 4). In HULECs treated with post-COVID sera, adding 1.2 μg/mL and 6 μg/mL gp120 to the incubation environment prevented the increase in tADA activity on the surface of HULECs (Figure 3C). In the TNFα-treated group, only the addition of 6 μg/mL gp120 counteracted the increase in cell-surface tADA activity (Figure 3D). In addition, after incubation of HULECs with recombinant SARS-CoV-2 spike protein S1 subunit (SP) in the presence of TNFα, we observed co-localization of immunofluorescence signal for CD26 and SP (Figure 3E). Simultaneously, pre-incubation with SP abolished TNFα inducement of cell-surface tADA activity in HULECs (Figure 3F).

Furthermore, adding 1.2 μg/mL gp120 to HULECs before the THP-1 cell adhesion assay resulted in less adhesion of immune cells after post-COVID serum stimulation (Figure 4). Interestingly, 1.2 μg/mL gp120 revealed a minor effect on THP-1 cell adhesion to the HULEC monolayer after TNFα stimulation (Figure 4).

## 4 Discussion

**FIGURE 5:**
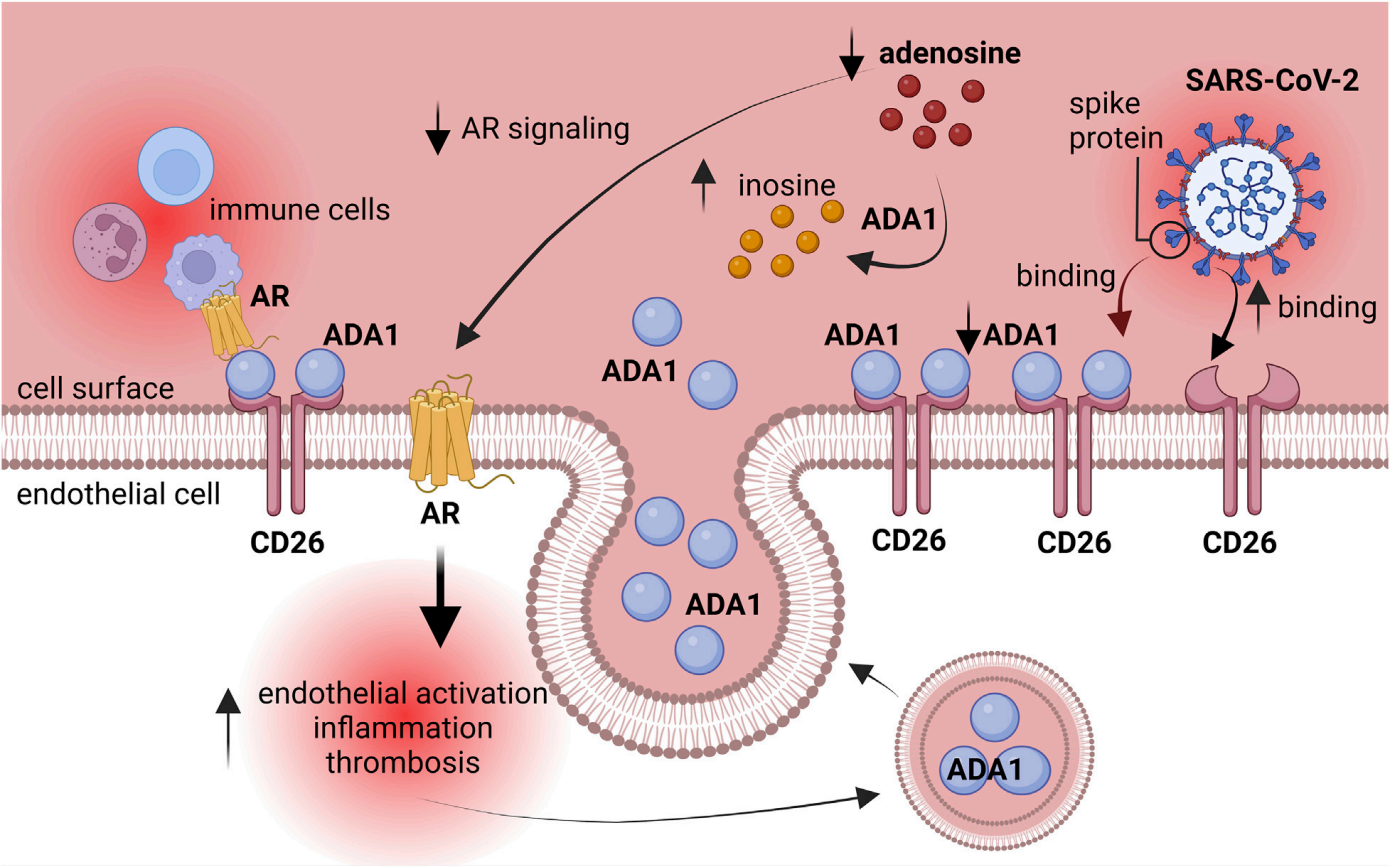
Explanatory figure. ADA1 competed with SARS-CoV-2 spike protein for CD26 binding. Activated adenosine deaminase silenced the protective effects of adenosine on the endothelium. AR–adenosine receptors, ADA1 – adenosine deaminase 1.

This study underlines the role of adenosine deaminase in cardiovascular complications after coronaviral infection. We revealed that human lung microvascular endothelial cells exposed *in vitro* to post-COVID patients’ sera upregulated cell-surface total ADA activity as well as the levels of CD26 and ADA1 proteins (Figure 5), while monocytes/macrophages treated with post-COVID sera, as well as PBMC isolated from post-COVID patients, revealed a pro-inflammatory phenotype related to the increased total ADA activity in cell extracts. Additionally, in the presence of post-COVID sera, we observed the enhanced adhesion of monocytes/macrophages to the endothelium, which may occur due to the CD26-ADA1 interaction.

This augmented adhesion of immune cells to endothelium can be due to the competition between ADA1 and SARS-CoV-2 for binding to the endothelial CD26 protein during the active phase of the infection (Raha et al., 2020; Radzikowska et al., 2020). This may alleviate the course of the disease, but in the long term, it can lead to the silencing of adenosine-dependent protective mechanisms due to increased adenosine degradation and prompting the interactions between immune cells and the endothelium via ADA1 non-enzymatic properties (Figure 5).

Since CD26 was considered an alternative receptor for SARS-CoV-2 binding, the anchoring of ADA1 to CD26 may disturb the cell entrance of the virus (Al-kuraishy et al., 2022). It has been shown that ADA competitively attaches to CD26, thereby preventing the binding of the Middle East respiratory syndrome coronavirus (MERS-CoV) spike protein domain to CD26 (Raj et al., 2014). In our study, recombinant SARS-CoV-2 spike protein S1 subunit abolished TNFα inducement of cell-surface tADA activity in endothelial cells. Simultaneously, we observed colocalization between membrane CD26 and virus spike protein. Thus, we assume that ADA1 can be recognized as a natural antagonist that blocks viral attacks, easing the severe course of a coronavirus infection. Still, at the same time, it may cause vascular complications after the infection due to adenosine signaling modulation and its role as an adhesion molecule. Indeed, we revealed that endothelial cells exposed to post-COVID patients’ sera switched into a pro-inflammatory phenotype, increasing their potential for monocyte/macrophage adhesion. Moreover, we have found that ADA1-CD26 interaction was critical for the adhesion of immune cells to endothelium, as gp120, the inhibitor of ADA binding to CD26, prevented monocyte adhesion induced by post-COVID sera and TNFα. Therefore, we suggest assessing *in vitro* functional tests using patients’ sera to predict late vascular complications and pro-inflammatory endothelial phenotype. It would also be valuable to extend this investigation to already vaccinated individuals, as our study primarily focused on and was limited to patients who had not undergone any form of COVID-19 prevention. Given that currently a significant percentage of the population has been vaccinated, their immune response to SARS-CoV-2 may differ. In addition, the administration of the competitors for the docking process of SARS-CoV-2 to CD26 may counteract the long-term adverse effects derived from ADA enzymatic and non-enzymatic properties, together with the prevention of viral entry. It was evidenced that treatment with sitagliptin, the inhibitor of CD26 enzymatic activity, reduced mortality in patients hospitalized for COVID-19 (Strollo and Pozzilli, 2020). It is plausible that the protective effects of CD26/dipeptidyl peptidase-4 inhibitors are mediated by the interference with the SARS-CoV-2-CD26 interaction (Raj et al., 2014; Lu et al., 2013).

In conclusion, this study highlights the role of ADA in endothelial inflammation after viral infections. The increase in ADA activity originating from endothelial and immune cells may favor vascular inflammation by silencing adenosine-dependent protective mechanisms. On the other hand, ADA1, via non-enzymatic properties, may act as an adhesion molecule between immune cells and endothelium by forming ADA1-CD26 complexes. We propose that ADA1, in competition with coronaviral particles for CD26 binding on endothelial cells during invasion, alleviates the course of the disease but instead favors long-term vascular complications after viral infections.

## Data Availability

The original contributions presented in the study are included in the article/supplementary material, further inquiries can be directed to the corresponding author.
